# Promises and Hurdles of Medical Tourism Development in the Russian Federation

**DOI:** 10.3389/fpsyg.2020.01380

**Published:** 2020-06-23

**Authors:** Arkady N. Daykhes, Mihajlo Jakovljevic, Vladimir A. Reshetnikov, Vasily V. Kozlov

**Affiliations:** ^1^N.A. Semashko Department of Public Health and Healthcare, F.F. Erisman Institute of Public Health, I.M. Sechenov First Moscow State Medical University, Sechenov University, Moscow, Russia; ^2^Department of Global Health Economics and Policy, University of Kragujevac, Kragujevac, Serbia; ^3^Institute of Comparative Economic Studies, Hosei University, Tokyo, Japan

**Keywords:** medical tourism, Russia, health policy, expenditure, medical care, emerging, services

## Abstract

**Background:** Development of medical tourism improves access to healthcare in countries where the necessary medical procedures are not available or accessible to its citizens. In the country of destination, medical tourism stimulates economic development and raises the quality of healthcare provided. There are both microeconomic and macroeconomic factors affecting the development of medical tourism. Microeconomic factors relate to the receivers and providers of healthcare. Macroeconomic factors relate to the government policy being implemented to support the development of medical tourism. This study aims to identify factors affecting the development of medical tourism in Russia.

**Methodology:** An expert survey of 36 heads of medical organizations in Russia was conducted to assess the problems that impede the development of the medical care system to foreign patients in Russia, as well as propose possible solutions. The degree of covariation among experts was calculated using the Kendall concordance coefficient.

**Results:** The experts gave consistent evaluation to numerous sets of problems that impede the development and proposed concrete measures for the development of inbound medical tourism in Russia. These measures ranged from microeconomic to macroeconomic approaches and were directed toward a holistic and coordinated development of medical tourism within Russia.

**Conclusion:** Based on the results, Russia has several micro- and macroeconomic competitive advantages and disadvantages in facilitating medical tourism. The study yielded a set of measures for the development of inbound medical tourism and the promotion of the export of medical services in the Russian Federation, which can be extended to other countries or parties who are seeking to develop medical tourism.

## Introduction

Medical tourism is a growing industry that facilitates travel to another country in pursuit of general medical or surgical care, which is not available or accessible in the country of residence. Development in technology and freedom of movement contribute to the growth of this industry (Crush, 2015; Cesario, 2018). Furthermore, significant growth of health spending worldwide has been documented in the emerging markets, led by BRICS (Jakovljevic et al., 2017). This landscape of strengthening legal framework and institutional capacities in these nations serves as a promise that they might 1 day become global hubs for medical tourism (Jakovljevic et al., 2019b).

Medical tourism allows patients to significantly decrease the cost and waiting time of medical care, while receiving higher or same quality care. Chuang et al. (2014) and Lovelock and Lovelock (2018) identified factors from the patient's side that affect the medical tourism industry: necessity of medical procedure, personal factors, factors related to the country of destination, and financial solvency. Currently, more than half of all users of medical tourism are women who seek medical services related to cosmetic or reproductive medical profiles (Cesario, 2018).

The development of medical tourism is important for improving the economy and the quality of healthcare in the country of destination. Recently, a number of studies took place, which were directed toward analysis of cost-effectiveness of medical tourism, overall community satisfaction, healthcare satisfaction, and attitudes toward medical tourism. These studies had proved that the above-mentioned factors directly affect local population's perception on the positive impact of medical tourism, which, in turn, affects the willingness to support the development of medical tourism in the country of destination (Sarantopoulos et al., 2015; Suess, 2018).

There have been several studies worldwide dedicated to analyzing organizational technologies and factors affecting medical tourism, the majority of which highlighted economic development from medical tourism as a beneficial impact for the society as a whole (Jakovljevic and Ogura, 2016).

During the evaluation of organizational effectiveness using the example of the medical tourism model in Malaysia, the need for the coordination of all elements of the healthcare system to foreign citizens and the existence of an established channel for the exchange of information were identified (Lee and Fernando, 2015). Heung et al. (2011) identified factors affecting the development of medical tourism in Hong Kong. Coordinated policies, regulations, government support, spreading expenses, issues with patient capacity, and the health needs of the local population are the main obstacles to the development of this type of tourism. Elimination of these barriers should be promoted by a new policy in the field of advertising. This approach should strengthen the government's policy to encourage investment in the medical tourism market, setting standards for the admission of foreign citizens for treatment. Thus, it could lead to the development of an entire range of medical services for foreign citizens in medical institutions.

The necessity of coordinated policy in relation to state organizers and medical professional community is also supported by a study conducted in Greece. It has revealed that private physicians often resist the development of the medical tourism segment in the country. They seem to be unsatisfied with the conditions for accepting foreign patients due to lower government prices for the provision of medical services to foreign patients, which reduces the salaries of doctors (Skountridaki, 2017).

Russia has a free and universal healthcare system for its citizens. All citizens participate in compulsory health insurance scheme provided by the government, which is financed from income tax. Both public and private medical organizations participate in the state insurance scheme. Private medical insurance schemes are available and provide treatment coverage in both private and public medical organizations. Majority of Russia's hospitals are publicly owned, divided into federal, regional, and municipal. Most of the healthcare volume is provided in public hospitals through a state insurance scheme. Foreign citizens are required to have private medical insurance scheme while in Russia.

Currently, the approximate size of the global medical tourism market is $439 billion annually, according to international payment systems (Destination Healthcare Guide, 2019). At the end of 2017, at least 11 million people went for treatment abroad. In accordance with the prediction that up to 4% of the planet's population would be within the range of living opportunities in order to receive medical care and in need of them, it is estimated that by 2025, global medical tourism market will increase to $3 trillion annually (Medical Tourism, 2019).

At the beginning of 2017, Russia was ranked 34th in the world offering medical services to foreign patients (Medical Tourism Index, 2018). The minister of healthcare of Russian Federation had stated that in 2017, Russia was visited by roughly 110,000 foreign patients for medical treatment (Skvortsova, 2018). Under Russia's strategic national development paradigm until 2024, inbound medical tourism needs to be increased to 1 billion USD annually, on estimate to 500,000 foreign patients treated annually. In comparison, Turkey had attracted 746,000 foreign patients for medical treatment in the same period (International Medical Travel Journal, 2017).

Currently, Russia accumulates roughly 70% of all inbound medical tourism within the Commonwealth of Independent States (CIS). Most patients come from the following regions: Central Asia (62% of patients), East European countries (32.2%), and Southeast Asia (5.2%). In public hospitals, inpatient care of the following profiles was in demand: oncology, ophthalmology, cardiology, oral and maxillofacial surgery, neurosurgery, traumatology, and orthopedics. In private medical organizations, less radical medical treatments were in demand: dentistry, gynecology, and reproductive technologies (MedRussia, 2019).

The authors of this study, having searched scientific literature on the subject, could not conclude that there is a single, united, and agreed methodology of organizing medical tourism in the world. Across the globe, different approaches in different countries with different healthcare systems are applied. Given the potential growth of this industry in the upcoming years and a deficit of scientific research in determining the best practices of organizing medical tourism, the authors believe that it is important to conduct research in this field.

The purpose of this study was to establish factors that contribute to the development of medical tourism in the Russian Federation and factors that impede the development of this area using expert assessments method. We seek to understand further ramifications of these complex relationships and how they refer to the health expenditure landscape in this vast region (Connell, 2013).

## Methodology

The following research focused on the scientific assessment of the modern Russian healthcare system in providing systematic medical care to foreign citizens in medical organizations of Sverdlovsk (Ekaterinburg) and Moscow regions, identifying main issues that impede its further development and possible solutions.

The research was conducted using the method of expert evaluations. The method consists of structured analysis of the problem by the chosen experts in the corresponding field, followed by a quantitative assessment of the experts' answers and evaluation of results. Kendall's coefficient of concordance was used to quantify the degree of agreement among the responders, Pearson fitting criterion was used to assess the significance of the differences in answers, and Friedman's non-parametric test was used for multiple comparisons of related samples (Glantz, 2011).

Experts were asked to evaluate on a five-point scale a set of measures aimed at optimizing the organization of medical tourism in the Russian Federation and to evaluate a number of aspects of its further development. A set of criteria were proposed regarding which experts to include into the research sample:

- Informed consent of the expert to participate in the study;
- Work experience in executive, administrative, or managerial position of 10 years or more;
- Experts' level of competence being average or higher (method of evaluation described below).

The number of experts depends on many factors and conditions. To determine the minimum number of experts required for this research, it was proposed to use the following formula (Mishin, 2003):

$$\begin{array}{r} n=\;0.5*\left(\frac{3}{e}+5\right) \end{array}$$

where:

n—the minimum number of experts required to participate in the research;

e—the margin of error of expert evaluation results (0 < e < 1).

With the proposed margin of error being (e) 0.05, the minimum number of experts required for valid results is 32. During the research, 36 expert samples were collected from the executive management of state medical organizations in the above-mentioned regions.

On the basis of the scientific study of management systems, the main criterion for selecting candidates for expert evaluation is the criterion of competence based on past performance (Gorshkov and Sheregi, 2009). To determine the level of competence, self-assessment method was applied.

The expert level of competence was evaluated on the basis of a self-assessment questionnaire, which allowed us to calculate the total competency index (k) of each expert. The average competency index for the entire expert group was also determined. The index was calculated on the basis of experts assessing their knowledge, experience, and forecasting abilities using a scale of “high,” “medium,” and “low.” The numerical values of the scales were 1, 0.5, and 0, respectively. In order to calculate the coefficient of expert competence, the following formula was used (Gorshkov and Sheregi, 2009; Sokolov et al., 2019):

$$\begin{array}{r} k=\;\frac{k_{1}+k_{2}+k_{3}}{3} \end{array}$$

where:

k_1_–the numerical value of the expert's self-assessed level of theoretical knowledge of the subject;

k_2_–the numerical value of the expert's self-assessed level of practical knowledge (experience) of the subject;

k_3_–the numerical value of the expert's self-assessed level of ability to predict future development of the subject.

The range of competency coefficient was from 1 (full competence, i.e., estimated values of the coefficients k1 = k2 = k3 = 1) to 0 (complete incompetence: k1 = k2 = k3 = 0).

The experts whose competency indexes were equal to or greater than the average were included in the research sample.

In order to quantify the degree of agreement between experts' answers, Kendall's coefficient of concordance was applied (Morozov, 2000): W < 0.3, the consistency of the expert's opinions being unsatisfactory; 0.3 < W < 0.7, the consistency of the expert's opinions being average; and W > 0.7, the consistency of the expert's opinions being high.

The calculation of Kendall's coefficient of concordance was done in several stages:

- The assembly of an expert panel group (number of evaluated factors *n* = 38; number of experts m = 36)
- Collection of experts' factors evaluation through a survey questionnaire and ranking the factors in a consecutive order;
- Compilation of a rank summary matrix based on a survey questionnaire followed by verification of the matrix.
- Degree of agreement assessment between experts' answers;
- Assessment of the statistical significance of the concordance coefficient using Pearson's consent criterion.

For visual representation of the final results, graphic geometric interpretations were used.

In addition, in order to assess the statistical significance of the differences in experts' answers within a certain question in the survey, the non-parametric Friedman test was used for multiple comparison of related samples. The Friedman test is a non-parametric statistical test that is a generalization of the Wilcoxon criterion and is used to compare objects with ranking according to individual measurement values. It is a non-parametric analog of ANOVA repeated analysis of variance (Glantz, 2011). Mean and standard deviations were calculated for experts' score answers. All calculations were carried out using IBM SPSS v.22.0.

All procedures performed in studies involving human participants were in accordance with the ethical standards of the institutional and/or national research committee and with the 1964 Helsinki Declaration and its later amendments or comparable ethical standards.

## Results and Discussion

Results on the expert's level of competence were the following:

- 86.1% of the respondents rated their level of theoretical knowledge on the subject as high;
- 75.0% of the respondents rated their level of practical knowledge (experience) on the subject as high;
- 69.4% of the respondents rated their level of ability to predict future development of the subject as high.

The average level of competency index for the formed expert panel group was satisfactory−0.86± 0.14.

### Q.1 Assessment of the Pressing Problems in Modern Russian Healthcare in Regard to Medical Services Provision to Foreign Patients

**Figure 1:**
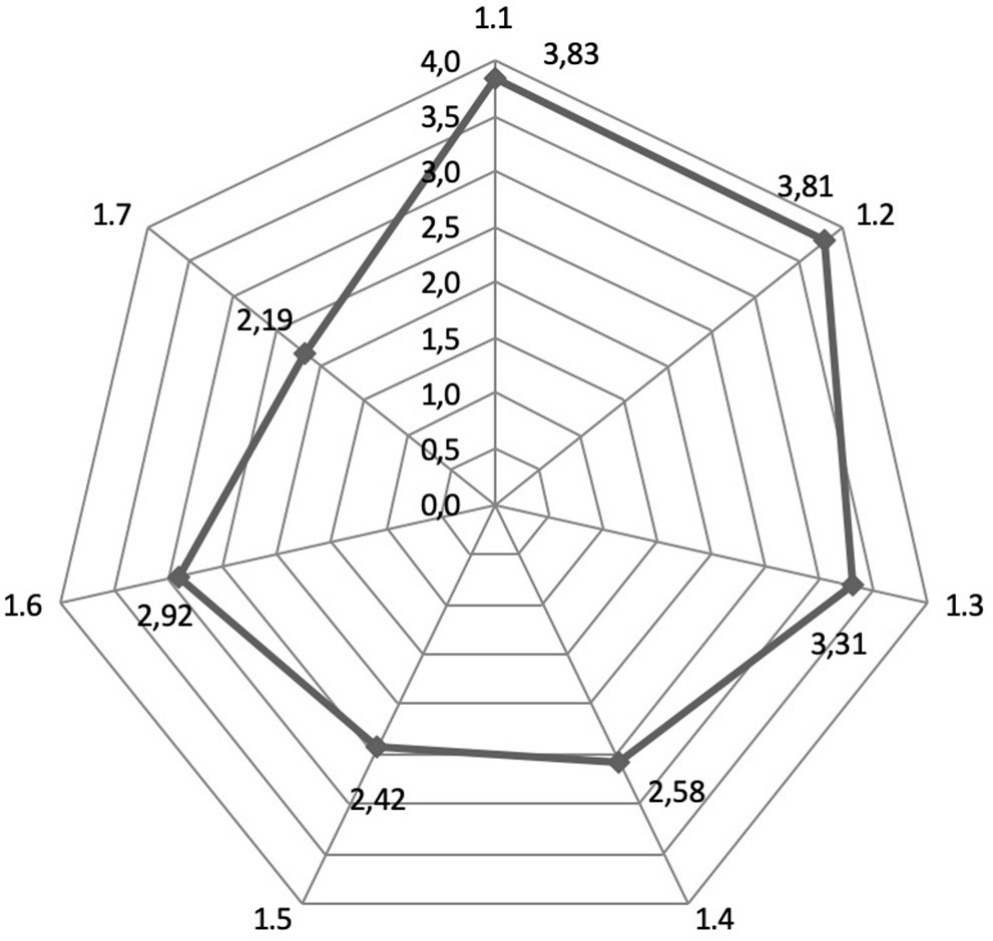
Assessment of the pressing problems in modern Russian healthcare in regard to medical services provision to foreign patients. (1.1—Lack of awareness of foreign patients; 1.2—Deficiency of medical personnel who speak foreign languages; 1.3—Deficiency of resources [staff, modern medical equipment, etc.]; 1.4—Deficiency of financial resources for foreign patients to pay for further treatment in case of complications; 1.5—The difficulty of obtaining and extending visas to foreign patients; 1.6—Lack of legal support for foreign citizens; 1.7—Lack of a state reporting system for providing medical assistance to foreign citizens).

Experts were asked to rate on a five-point scale the importance of the most pressing problems in modern Russian healthcare system, which hinder or impede the development of a systematic provision of medical services to foreign citizens; experts were also able to give an open-ended answer. The results are represented in Figure 1.

The highest rating in terms of significance (average = 3.83; s.d. ± 1.30) was applied to the problem of insufficient awareness of foreign patients about opportunities to receive good quality medical services in the Russian Federation. The problem of shortage of medical personnel who speak foreign languages in domestic clinics was also present (average = 3.81; s.d. ± 1.50). The third identified problem was a shortage of resources in the Russian medical organizations (average = 3.31; s.d. ± 1.14).

Among the most frequent problems formulated by the experts in the open-ended answers were the lack of developed transport logistics for delivering foreign citizens to medical centers (11.1% of the expert panel group), the lack of money for international quality certification of Russian medical organizations (8.3% of the expert panel group), and the lack of supporting services that take into account the nationalities of the patients (food, accommodation, leisure, etc.; 8.3% of the expert panel group).

### Q.2 Necessary Basic Measures to Create a System of Export of Medical Services to Foreign Patients in the Russian Federation

The generalized description of experts' answers to an open question about the necessary basic measures for creating a system of export of medical services to foreign patients in the Russian Federation is as follows.

Measures in the field of providing medical organizations of the Russian Federation with qualified personnel and material and technical resources were mentioned most frequently (25% of the experts). This category included introducing modern technologies, guidelines, and standards for the provision of medical services, improving the material and technical basis of medical organizations to expand the range of services, and studying and using world standards of service in the export medical services market.

Measures to increase the level of awareness of foreign citizens, including those related to the use of modern information and communication technologies, were mentioned in the second rank (22.2% of the experts). This category included information support for the provision of services to foreign citizens on the websites of medical organizations, posting of information on foreign language websites about the possibility of providing certain types of medical services in the institutions of this region, the work of call centers to inform patients, the creation of mobile applications, and online counseling for an initial selection of foreign citizens.

Organizational measures were mentioned in the third rank (16.7% of the experts). This category included the creation of an organizational structure to support the export of medical services as a coordinating center at the federal level in the format of an autonomous non-profit organization and the development of road maps for the implementation of regional projects for the development of international tourism.

### Q.3 Competitive Advantages of Medical Organizations of the Russian Federation for the Formation of a Steady Flow of Foreign Medical Tourists

The description of experts' answers to an open question about competitive advantages of medical organizations of the Russian Federation for the formation of a steady flow of foreign medical tourists is given as follows.

Availability of advanced medical procedures at a relatively low cost of treatment was mentioned most frequently (55.6% of the experts).

A high level of material and technical equipment of medical organizations and the qualifications of medical personnel were mentioned in the second rank (47.2% of the experts).

Features of the geographical location of the Russian Federation, in particular, a large number of border countries and proximity to Central Asia, as the most promising region for medical tourism were mentioned in the third rank (16.7% of the experts).

### Q.4 Assessment by Experts of Ways to Promote Medical Tourism to Attract Foreign Patients

**Figure 2:**
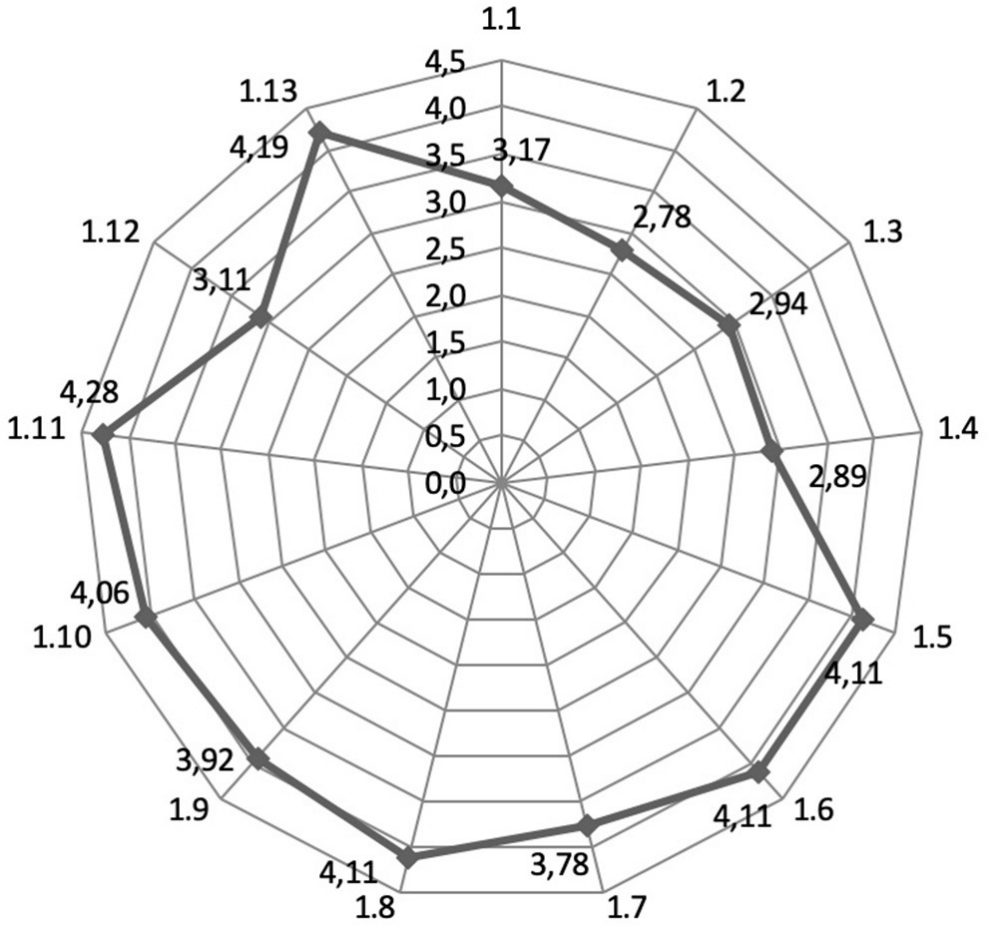
Assessment by experts of the ways to promote medical tourism to attract foreign patients to receive medical services in the Russian Federation [1.1—Advertising on television; 1.2—Advertising on the radio; 1.3—Advertising in the print media; 1.4—Outdoor advertising; 1.5—Promotion of the medical website organizations in search engines Yandex, Google, etc.; 1.6—Social networks (Vkontakte, Twitter, Facebook, etc.); 1.7—Development of the VHI sector in foreign countries; 1.8—International cooperation at the state level; 1.9—Participation in international exhibitions; 1.10—Participation in international conferences; 1.11—Recommendations of doctors and partner organizations abroad and in the Russian Federation; 1.12—Work with messages sent to the website of a medical organization; 1.13—Recommendations of patients who have previously been treated in medical organizations of the Russian Federation].

Experts were asked to rate on a five-point scale the ways to promote medical tourism to attract foreign patients in order to receive medical services in the Russian Federation. The results are presented in Figure 2.

The highest rating in terms of significance (average = 4.19; s.d. ± 1.26) was obtained for using the recommendations of doctors and partner organizations abroad and in the Russian Federation (*p* < 0.05 by Friedman test). Using the recommendations of patients who have already been treated in the clinics of the Russian Federation in the framework of medical tourism was recognized as in the second rank of significance (average = 4.28; s.d. ± 1.03). The third rank of significance (average = 4.11 points; s.d. ± 1.29) was divided by such measures as promoting a medical organization's website in search engines (Yandex, Google, etc.), using social networks (Vkontakte, Twitter, Facebook, etc.), and promoting medical tourism through international cooperation at the state level.

### Q.5 Assessment by Experts of Various Forms of Payment by Foreign Citizens for the Provision of Medical Services

The description of experts' answers to an open question about which form of payment by foreign citizens for the provision of medical services is the most preferable is presented as follows.

The experts recognized the use of cash payment by the patient himself or herself with the mandatory presence of his or her voluntary medical insurance policy as the most preferred payment method (69.4% of the experts).

Payment by the patient himself in cash was mentioned by the experts in the second rank (22.2%); 13.9% of the experts believe that payment for medical services of foreign citizens should occur only within the framework of voluntary medical insurance (third rank).

### Q.6 Organizational and Legal Decisions Necessary for the Development of Medical Services Export in the Russian Federation

The description of experts' answers to an open question about the necessary organizational and legal solutions for the development of the medical services export system in the Russian Federation is provided as follows.

The need to improve the legislation at the federal and regional levels of the Russian Federation was mentioned most often (33.63% of the experts).

The visa facilitation for foreign citizens wishing to receive medical care in the Russian Federation was mentioned in the second rank (13.9% of the experts).

The need to improve the system for providing paid medical services and to develop the procedure for their provision was mentioned in the third rank (5.6% of the experts).

### Q.7 Assessment by Experts of the Prospects of Geographical Regions for the Development of Inbound Medical Tourism in the Russian Federation

**Figure 3:**
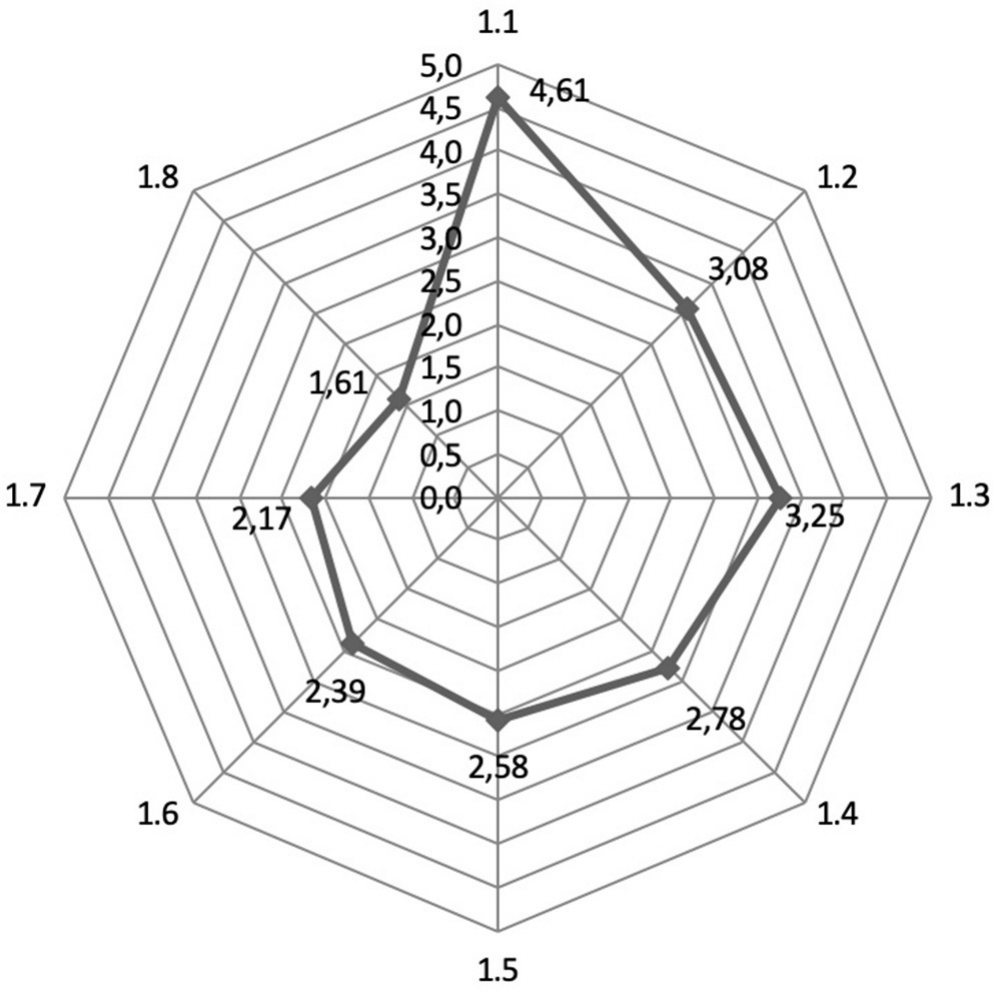
Assessment by experts of the prospects of geographical regions for the development of inbound medical tourism in the Russian Federation (1.1—Commonwealth of Independent States; 1.2—Eastern Europe; 1.3—East Asia; 1.4—Southeast Asia; 1.5—Middle East; 1.6—Africa; 1.7—Western Europe; 1.8—North America).

The experts were asked to rate on a five-point scale the prospects of geographical regions for the development of inbound medical tourism in the Russian Federation. The results are presented in Figure 3.

The experts recognized the neighboring countries (Commonwealth of Independent States) as the most promising region in terms of attracting foreign patients (average = 4.66; s.d. ± 0.69), which, according to the Friedman test, was statistically significantly different from the estimates of all other regions (*p* < 0.05). The second- and third-ranking places that experts gave are East Asia (average = 3.25; s.d. ± 1.40) and Eastern Europe (average = 3.08; s.d. ± 1.44), respectively, as promising areas for attracting medical tourists.

### Q.8 Assessment by Experts of the Measures to Improve the Development of Inbound Medical Tourism and to Promote the Export of Medical Services in the Russian Federation

**Figure 4:**
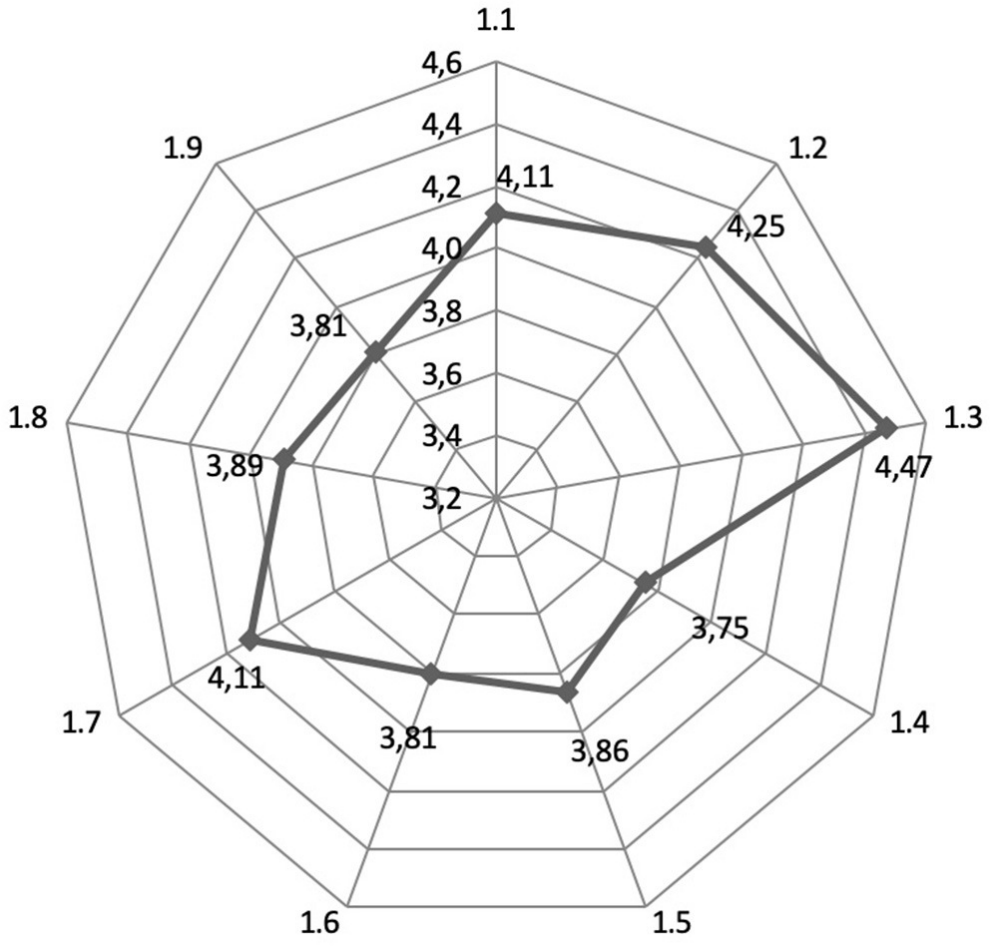
Assessment by experts of the measures to improve the development of inbound medical tourism and to promote the export of medical services in the Russian Federation [1.1—Solving the problem of a medical visa facilitating for medical tourists; 1.2—International certification and accreditation of medical centers; 1.3—Using the latest treatment technologies in combination with an international level service based on the principles of “everything for the patient”; 1.4—Developing and implementing educational programs for the study of foreign languages among medical staff; 1.5—Coordinating body to promote the export of medical services; 1.6—Training of medical staff in international service skills; 1.7—Information support (creation of a specialized site for advertising medical services); 1.8—Economic benefits for medical tourism organizations; 1.9—Formation of packages of integrated services including both medical and non-medical services for the support of foreign nationals].

The experts were asked to rate on a five-point scale the system of measures to develop inbound medical tourism and to promote the export of medical services in the Russian Federation. The results are presented in Figure 4.

The experts recognized the use of the latest treatment technologies in combination with an international level service based on the principles of “everything for the patient” as the most important measure (average = 4.47; s.d. ± 0.94). The experts recognized international certification and accreditation of medical centers (average = 4.25; s.d. ± 1.30) as the second group of measures in significance, and information support (creating a specialized site for the promotion of medical services; average = 4.11; s.d. ± 1.19) as the third group in significance.

Experts focused on the need to offer foreign medical tourists a wide range of medical services from small manipulations to complex high-tech procedures and comprehensive “all inclusive” packages due to the different financial capabilities and different life situations of the alleged patients in the open part of the question.

### Q.9 Assessment by Experts of the Export Potential of Medical Organizations in the Russian Federation

The question about assessing the export potential of medical organizations was directed only to experts who hold executive management positions in medical organizations (*n* = 18).

An increase in the number of treated patients by 10–20% was mentioned most often (55.6% of the experts) as the export potential of a medical organization, which it is able to realize without prejudice to the state guarantee program. An increase in the number of treated patients by 20–40% was mentioned in the second rank (27.8% of the experts), and an increase in the number of treated patients by 40–60% was mentioned in the third rank (5.6% of the experts). In only one case (5.6%), the expert (executive manager) answered that his organization lacked export potential.

### Q.10 Assessment by Experts of the Amount of Investment in Medical Organizations Required to Obtain International Certification

The description of experts' answers to an open question about the amount of investment in medical organizations of the Russian Federation required to obtain international certification is given as follows. This question was directed only to experts who hold executive management positions in medical organizations (*n* = 18).

The majority of the experts (61.1%) indicated that significant investments were required first of all for major repairs, equipment purchases, and other large expenses; 22.2% of executive managers noted that international certification requires investments for staff training, cosmetic repairs, and other minor expenses; 16.7% of executive managers believe that their organization is ready for international certification and only minor investments are required.

## Conclusion

There are a few pressing issues in modern Russian healthcare system that hinder or impede the development of the provision of medical services to foreign citizens (Goroshko and Pacala, 2018; Jakovljevic et al., 2019a); together with the results from the panel of experts, they can be summarized as follows:

- Insufficient awareness of foreign patients about the possibility of receiving high-quality medical services in the Russian Federation.
- Deficit in domestic clinics of medical personnel who speak foreign languages.
- Underdeveloped tourism services and infrastructure.
- Weak marketing abroad (bad packaging) of tourism opportunities in Russia.

**Figure 5:**
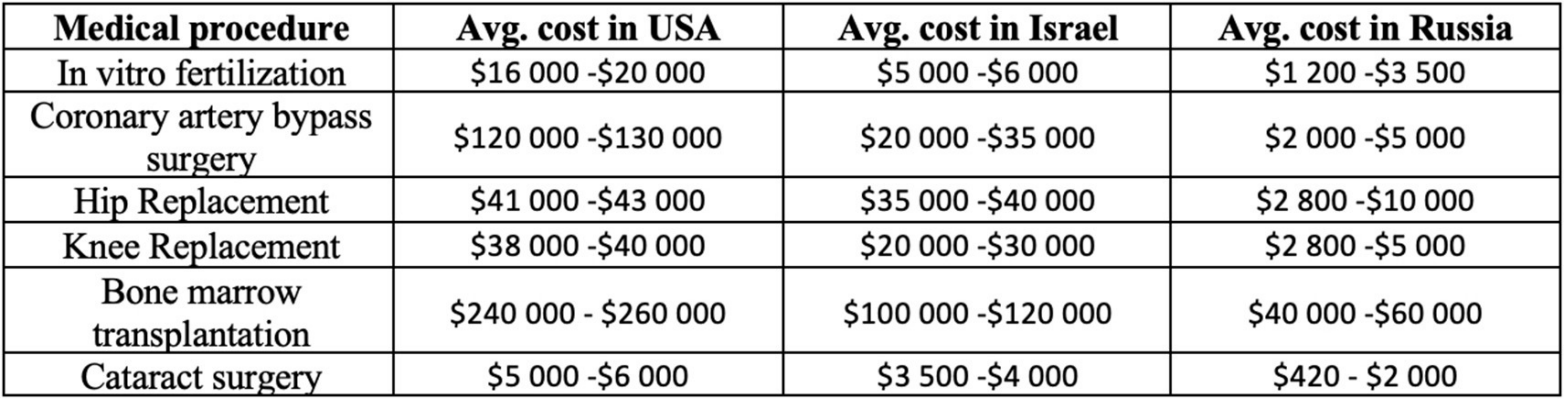
Price comparison of medical procedures between major hospitals in the USA, Israel, and Russia (Klimin et al., 2019).

However, the Russian healthcare system has a strong competitive advantage in the development and provision of medical tourism (Reshetnikov et al., 2020):

- Ability to offer diverse and advance medical services;
- Demand from CIS countries with less developed healthcare system;
- Additional demand from numerous work migrants present in Russia;
- Economic viability of obtaining advanced medical services by foreign medical tourists in Russian medical organizations, due to significantly lower prices in comparison to leading countries within medical tourism (Figure 5).

According to experts, CIS countries, Eastern Europe, and East Asia are the most promising regions in terms of attracting foreign patients to receive medical services in the Russian Federation (Reshetnikov et al., 2019).

The system of providing medical services to foreign patients in Russia is at the initial stage. In order to increase the flow of medical tourists to Russia, necessary actions to promote medical tourism in Russia need to be taken. These include the formation of partnerships with medical organizations abroad in order to diagnose, select, and rehabilitate patients; active advertisement of the experiences of patients being treated in Russia; targeted promotion of Russian medical organizations in internet search engines in the country of patients' origin; and targeted promotion in social networks of individuals seeking cost-efficient medical care (Tsymbal and Consultant, 2014).

To increase the efficiency of providing medical services to foreign patients, necessary basic measures need to be taken. Russian medical organizations are required to increase competencies of medical personnel dealing with foreign patients by demanding these personnel to have the knowledge of foreign languages (primarily English) or at least facilitate a system where the foreign patient is accompanied by a translator. Additionally, further improvement in capital expenditure would increase the level of service provided (Jakovljevic et al., 2008). A series of marketing steps in the targeted country would increase foreign patients' awareness of opportunities in medical tourism in Russia.

The experts gave good assessment with a high degree of consistency to the following set of measures for the development of inbound medical tourism and the promotion of the export of medical services in the Russian Federation:

1. The use of the latest treatment technologies in combination with an international level service based on the principles of “everything for the patient”;
2. International certification and accreditation of medical centers;
3. Measures of information support for the promotion of inbound medical tourism using the capabilities of the Internet;
4. Facilitated obtaining a medical visa for medical tourists;
5. Economic privileges for organizations of the medical tourism system;
6. The creation of a single coordinating body to promote the export of medical services;
7. Training of medical staff in service skills at an international level;
8. The formation of packages of comprehensive services, including both medical and non-medical services to accompany foreign citizens;
9. The development and implementation of educational programs for the study of foreign languages among medical staff.

This brief research report identifies promises and hurdles of medical tourism development in the Russian Federation. This brief research report also identifies regions that are most promising in attracting foreign patients for treatment in Russia, lays out general marketing steps, and delivers micro- and macroeconomic measures necessary to increase the quality of care and flow of foreign patients to Russia. Complete research results and their concrete scientific findings will be presented once the research is concluded.

## Data Availability Statement

The raw data supporting the conclusions of this article will be made available by the authors, without undue reservation.

## Ethics Statement

Ethical review and approval was not required for the study on human participants in accordance with the local legislation and institutional requirements. Written informed consent to participate in this study was provided by the participants.

## Author Contributions

AD: data collection, literature review, data compiling, data analysis, and main text formulation. MJ: development of research's concept, expert editorial guidance, and analysis and interpretation of results. VR: research framework development and guidance, expert editorial guidance, and analysis and interpretation of results. VK: development of research methodology and data analysis. All authors contributed to the article and approved the submitted version.

## Conflict of Interest

The authors declare that the research was conducted in the absence of any commercial or financial relationships that could be construed as a potential conflict of interest.
